# The Role of *Helicobacter pylori* Proinflammatory Outer Membrane Protein and Propolis in Immunomodulation on U937 Macrophage Cell Model

**DOI:** 10.31661/gmj.v9i0.1687

**Published:** 2020-12-08

**Authors:** Hengameh Soudi, Tahereh Falsafi, Sara Gharavi, Mohaddeseh Mahboubi

**Affiliations:** ^1^Microbiology Department, Faculty of Biological Sciences, Alzahra University, Tehran, Iran; ^2^Biotechnology Department, Faculty of Biological Sciences, Alzahra University, Tehran, Iran; ^3^Medicinal Plants Research Department, Research and Development, Tabib-Daru Pharmaceutical Company, Kashan, Iran

**Keywords:** Helicobacter pylori, IFN-γ, IL-4, Immune Response, OipA, Propolis

## Abstract

**Background:**

Regarding the important role of proinflammatory outer membrane protein (OipA) in the pathogenesis of *Helicobacter pylori* infection and immunomodulatory activity of propolis, we aimed to evaluate the immunogenicity effect of a purified recombinant OipA protein and propolis in the induction of two cytokines, interferon-gamma (IFN-γ) and interleukin-4 (IL-4), in a macrophage cell model.

**Materials and Methods:**

The recombinant protein used in the present study corresponding to the *oipA* expressing a 34-35 kDa protein. OipA protein was purified by Ni-NTA affinity chromatography. The purified OipA protein (2.5- 40 μg /mL) and the propolis ethanolic extract (5-40 μg/mL) were incubated with phorbol 12-myristate 13-acetate-treated human myelomonocytic cell line U937 cells. IL-4 and IFN-γ levels were measured after 48 hours of incubation using enzyme-linked immunosorbent assay.

**Results:**

The amounts of IL-4 and IFN-γ were significantly increased. The optimum concentration of OipA for the secretion of IL-4 was 5 μg/ml (P<0.0001). At higher concentrations, the amount of IL-4 diminished until suppression at 40 μg/mL. The optimum concentration of propolis, resulting in the most significant increased secretion of both IL-4 and IFN-γ was 40 μg/mL (P=0.0001 and P=0.0004).

**Conclusion:**

We found that an OipA concentration of 10 μg/mL was more effective for IFN-γ production; however, it was not effective for the high production of IL-4. Therefore, it is postulated that the OipA could mainly induce a Th1 response through the production of IFN-γ. We also observed propolis’s capability to induce IFN-γ production; however, the effective concentration for this was the same as for IL-4. Therefore, as an adjuvant, proper concentration of propolis is required for OipA to give the optimum response.

## Introduction

*H elicobacter pylori* colonize the gastric mucosa of half the world’s population. Its infection is associated with gastrointestinal diseases, including gastritis, peptic ulcer disease, gastric adenocarcinoma, and gastric mucosa-associated lymphoid tissue lymphoma (MALT) [1]. The current formulation of antibiotic therapy is the only way to eradicate *H. pylori* infection. However, in many geographic regions, successful eradication has failed due to the emergence of resistant strains. Moreover, in these geographic regions, reinfection contributes to the lifelong infection impact [2]. Therefore, other therapeutic or prophylactic methods are needed to resolve the problems associated with *H. pylori* infection. Among the most important virulence properties of *H. pylori* is its adhesion to gastric epithelial cells, which may be the first important step in the pathogenesis of infection. Among a few important adhesins of *H. pylori*, outer inflammatory protein A (OipA) is an important one, which plays a role in the particular interaction with the host cell membranes and favors the production of a proinflammatory cytokine, interleukin -8 (IL-8) [3]. It is assumed that Th1 effectors cells mediate protection against *H. pylori* infection through the production of proinflammatory cytokines, interferon-gamma (IFN-γ) as key cytokine and also tumor necrosis factor (TNF)-α and –β [4,5]. Numerous *H. pylori* antigens have been investigated as vaccine candidates to find an efficient antigen [6]. Among them, *H. pylori* OipA, which is an important adhesin of *H. pylori*, may be an important antigen. This outer membrane protein may trigger the production of the proinflammatory cytokine, IL-8, by unique interactions with the host cell membranes, resulting in increased inflammation-causing peptic ulcers and other infections [3,7]. Recent studies have demonstrated the role of OipA in the induction of mucosal cytokines such as IL-1, IL-17, and TNF-α [8]. The functional *OipA* gene is expressed into a 33-35 Kilo dalton (kDa) protein and is regulated by the slipped-strand repair mechanism, which changes the number of CT dinucleotide repeats in the 5’-region of the *Oip*A gene. Therefore, a switch “on” status is functional, and a switch “off” is nonfunctional [9,10]. Yamaoka *et al*., as the first important investigators in this field, have reported that functional OipA is a 34-kDa protein [6] and the complete *Oip*A gene, which encodes this protein, is 930 bp in length. The recombinant protein used in the present study is almost identical to this reported clone. Although experimentation of OipA protein may be important in protecting the host against *H. pylori *infection, obtaining the optimum results will depend on the association of this antigen with a potent adjuvant, aiming to increase its immunogenicity. It is also essential to have effective protection, which may be through efficient activation of specific effectors of the immune system, such as cytokines mediating the operative T cell responses against *H. pylori*. Even though many adjuvants of several origins have been evaluated, most commercial vaccines continue to rely on aluminum salts. Also, the use of natural adjuvants instead of traditional chemicals to prevent their side effects may be promising in the vaccination process. Among them, propolis may be a suitable candidate because its adjuvant properties have been demonstrated in the vaccination of mice models [11]. Propolis or “bee glue” is a resinous compound collected by honeybees from plant’s flowers. It is a natural substance that harvested by honeybees from different parts of plants such as shoots, buds, and resinous exudates [12-14]. Chemically complex, propolis is composed of more than 300 different substances depending on the regions [14,15]. In addition to many different biological and pharmacological properties of its different preparations, it has been reported to have immunostimulator and immunomodulation activities [16-18]. Therefore, propolis, with these properties can be an experiment as an adjuvant toward OipA as an antigen. This study aimed to evaluate the immunogenic effect of a purified recombinant OipA protein as selected antigen in a macrophage cell model and propolis as a novel adjuvant, since; in this model, we could evaluate more practically the immunogenic effects of OipA as well as the adjuvant potentially of propolis, with determining the amount of key cytokines production by the macrophage.

## Materials and Methods

###  Preparation and Identification of Recombinant OipA Protein

The used strain to construct a functional clone for the *OipA* expression under full-length OipA protein has been obtained from a patient demonstrating severe active gastritis [19]. This functional recombinant clone containing the *OipA* gene was constructed in a previous study [19]. Briefly, a clinical *H. pylori*-isolate demonstrating high expression for an outer membrane protein (OMP) with an apparent Molecular weight (MW) of 33-35 kDa was selected. The *OipA* specific primer was designed according to the *OipA* gene sequences from the B8 strain. The purified PCR-product was sequenced and submitted to Gene Bank (gb/KJ816695.1). The pET-28a plasmid and *Escherichia coli *DH_5_α were used for cloning and transformation. The recombinant plasmid was transferred to *E. coli* BL21 (DE3) [19]. Induction of OipA recombinant protein was done in Luria broth (LB) broth by 1 mM isopropyl β-D-1-thiogalactopyranoside (IPTG; Sigma Aldrich, Germany). OipA protein was purified by Ni-NTA affinity chromatography (Takara, Japan) using a hybrid denaturation method on-column resolubilization method. Briefly, the cell pellet from the induced culture was collected, washed and dissolved in binding buffer (100 mmol/L NaH2PO4, 10 mmol/L Tris-HCl, and 8 mol/L urea; pH: 8), and after removing insoluble materials by centrifugation, 1 ml Ni-NTA Agarose (50%) was added to every 5 ml of the clear lysate and was placed on a rotatory shaker for 1 h (batch purification). The mixture was loaded onto the column and washed by denaturing wash buffer (100 mmol/LNaH2PO4, 10 mmol/L Tris-HCl, and 8 mol/L urea; pH: 6.3) and also denaturing wash buffer plus 8 mol/L urea (pH: 5.9). Urea removal and resolubilization of the recombinant protein were obtained by washing the resin with a series of wash buffers by decreasing urea concentrations (50 mmol/L NaH2PO4, 300 mmol/L NaCl, 5% ethanol, pH 8) containing 8, 6, 4, 2, 1, and 0 mmol/L urea, which were added by one volume to the column. Recombinant OipA was eluted from the column by elution buffer (250 mmol/L imidazole, 50 mmol/L NaH2PO4 and 250 mmol/L NaCl, pH: 8). Purified fractions were analyzed by SDS-PAGE and then pooled and dialyzed against 20 volumes of PBS at 4°C/ overnight. The concentration of OipA protein was evaluated by the Bradford test, and the identification of the purified protein was confirmed by Western blotting using an anti-His-tag monoclonal antibody (Sigma Aldrich, Germany). Alternatively, this recombinant protein was identified using a rabbit nonspecific antibody obtained against OipA according to the previously reported protocol [19]. In the present study, Polymyxin B sulfate (20 μg/mL) was also added to remove lipopolysaccharide (LPS) from the solution and the level of LPS was measured by Limulus Amebocyte Lysate Assay kit (Gen Scrip, USA). This step of purification will suppress the stimulating and/or toxic effects of LPS in cell culture.

###  Propolis Preparation

An Iranian Propolis sample prepared by Sepahan ASAL, Isfahan (Iran), from colonies of honeybees located in Isfahan, was evaluated as a candidate for its role in inducing the immune response and as a candidate for a potential natural adjuvant. For this evaluation, 1g of propolis was ground and mixed with 50 mL of ethanol (70%), then was stirred at room temperature for 24 h; the extract was filtered and the solvent was evaporated under vacuum at 50 °C until it was dried [23].

###  Cell Culture

The human myelomonocytic cell line U937 (ATCC CRL-1593.2) obtained from Pasteur Institute (Tehran, Iran) was grown in RPMI 1640 (Gibco, UK) supplemented with nonessential amino acids and 5% fetal bovine serum (FBS; Gibco, UK), 5 U/mL penicillin, and 5 mg/mL streptomycin at 37°C in 5% CO2 to a density of 5x10^5^ cells/mL in RPMI medium. For all experiments, the U937 cells, which are not adherent cells to culture plates in its natural state, were differentiated into the macrophages by adding phorbol 12-myristate 13-acetate (PMA; Sigma-Aldrich, Germany) at a final concentration of 100 nM, 48 h before the infection. The purified protein was added to the treated U937 cells by PMA at specific concentrations (2.5, 5, 10, 20, and 40 μg /mL) to experiment with the effects of OipA protein incubation with the macrophages. In all experiments, PBS was employed as a negative control. Cytokines (IL-4 and IFN-γ) concentrations were determined after 48 hours of incubation. The ethanolic extract was added to the PMA treated U937 cells at specific concentrations (5, 10, 20, and 40 μg /mL) to experiment with the effects of propolis on the macrophage cells. Cytokines concentrations were determined after 48 hours of incubation.

###  Measurements of Cytokines

 IL-4 and IFN-γ (R&D systems, USA) levels were measured in the supernatant of treated cells using enzyme-linked immunosorbent assay (ELISA) and standard method (R&D systems, USA).

###  Statistical Analysis

 Ordinary one-way ANOVA and multiple comparisons were used to compare the differences between groups. The graphs were drawn with Graph Pad software (Graph Pad Prism 8, GraphPad Software Inc., California, USA). A P-value less than 0.05 was considered as the significant level.

## Results

###  Characterization of Recombinant OipA Protein

**Figure 1:**
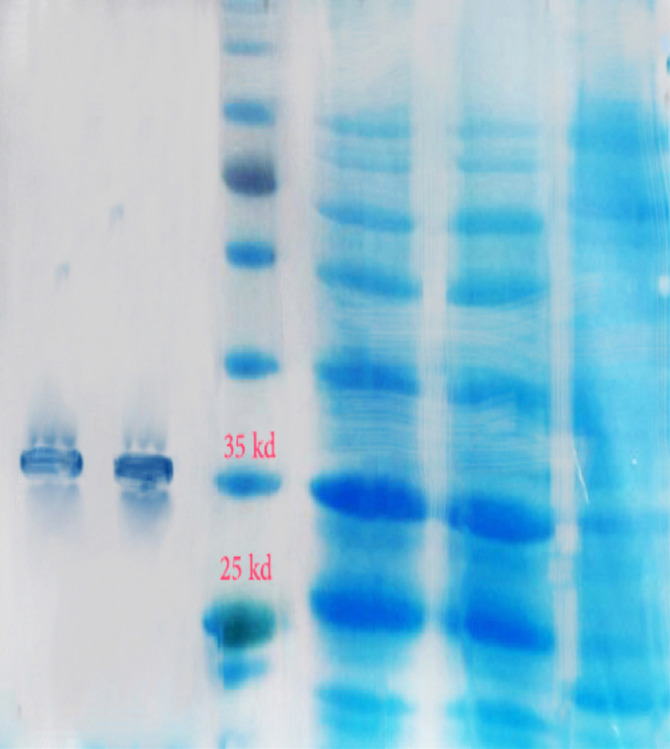
The Coomassie blue-stained 12.5% SDS-PAGE gel, showing induction of the expressed OipA protein; right to left: **1**: non-induced OipA, **2:** and **3:** induced OipA with 1 mmol/L IPTG, **4:** standard low protein size marker (10-245 kDa), **5** and **6:** purified OipA (34-35 kDa).

**Figure 2:**
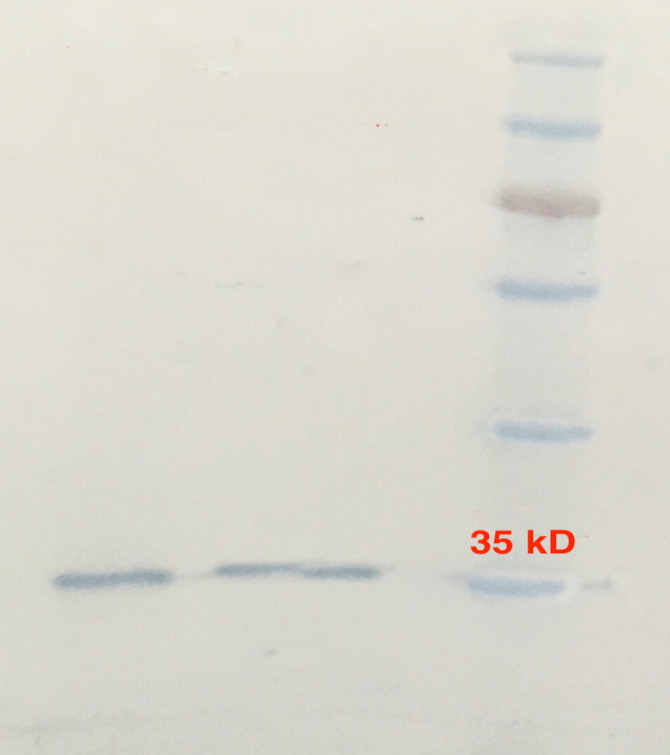
Western blot analysis using the OipA antibody, demonstrating a single 34-35 kDa protein on nitrocellulose paper.

The sequence of the *oipA* gene and the MW of the purified recombinant OipA protein consisted of 924 bp and 33-35 kDa, respectively. Its identity with other published *oipA* genes was 92-96%; the highest identity was observed with a Mexican *oipA* clone obtained from an *H. pylori *strain associated with severe symptoms [19]. Production of recombinant OipA protein was induced with optimum concentration (1 mmol/L) of IPTG. Recombinant OipA protein was purified by His-tag monoclonal antibody. The protein was identified in 12.5% SDS-PAGE gel (Figure-1). The purified OipA protein was detected by the anti-His-tag monoclonal antibody in Western blotting and confirmed the presence of 34-kDa protein (Figure-2). The amount of LPS after its blockage by Limulus Amebocyte Lysate Assay kit was less than 0.25 EU/mL.

###  Morphology of Host Cells Before and after PMA Treatment

**Figure 3:**
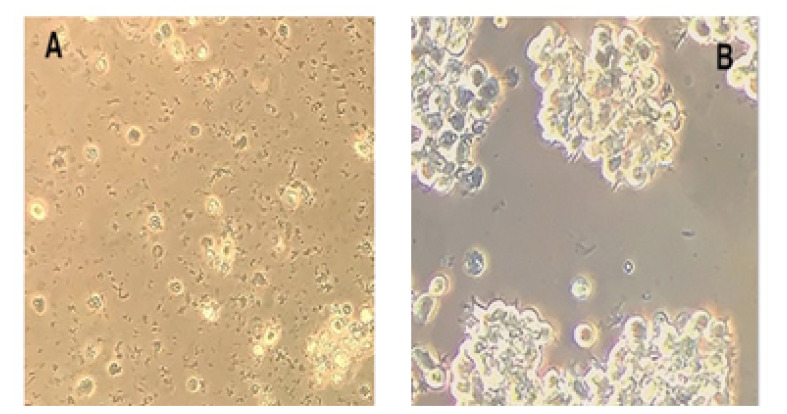
U937 cell cultures. A: U937 cells before PMA treatment. B: Adherent PMA activated U937 after 48 h PMA treatment.

 In standard culture medium, U937 cells typically had a smooth and round shape and were not adhere to the culture plates. Following PMA treatment, these cells became adhesive on the culture plates by forming cell clusters, extended pseudopodia, and almost differentiated into the adherent macrophage (Figure-3).

###  Measurement of IL-4 and IFN-γ in OipA and Propolis Treated Macrophage Cells

**Figure 4:**
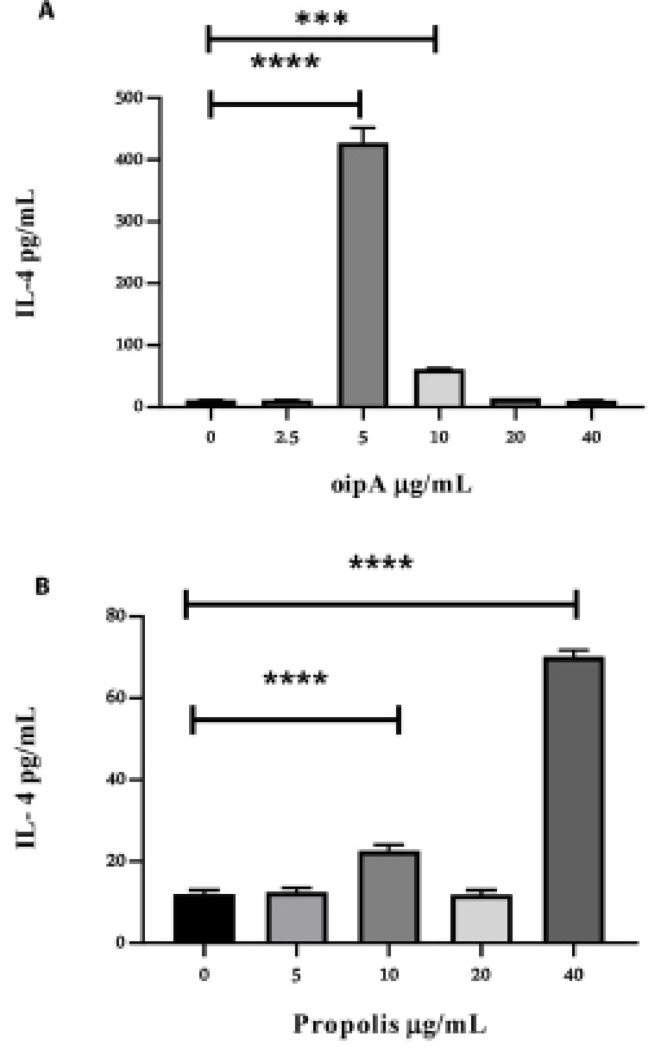
IL-4 concentrations after propolis (A) and OipA (B) treatment. The concentration of IL-4 (pg/mL) was expressed as a mean of three independent experiments ± SD. Statistically significant differences with the negative control are indicated as ****(P<0.0001); ***(P=0.0004).

**Figure 5:**
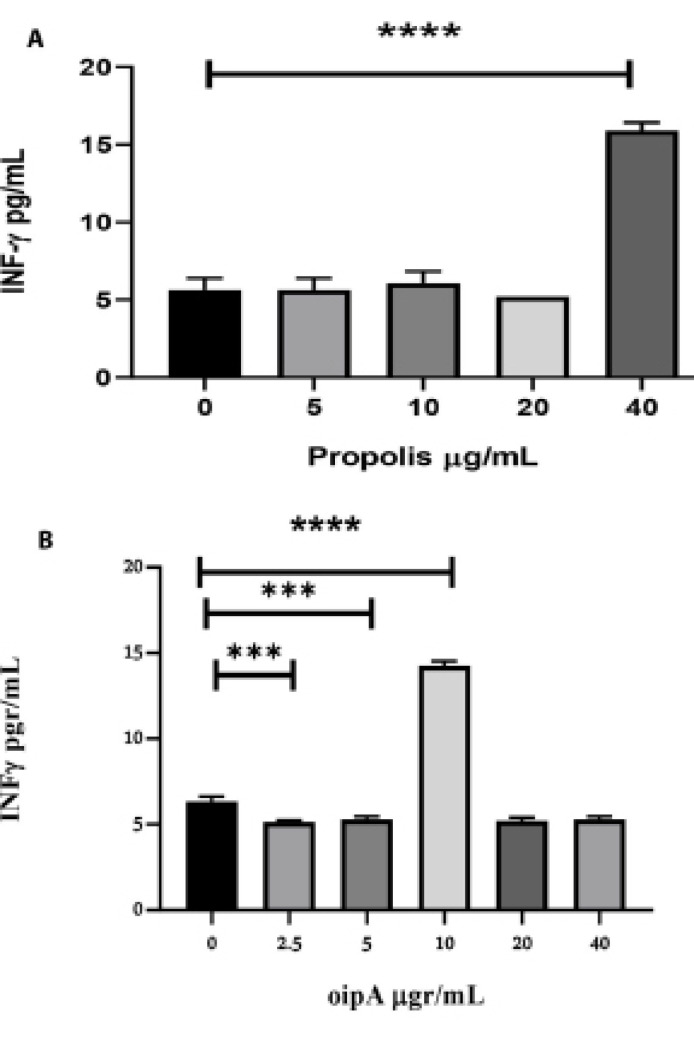
IFN-γ concentrations: Propolis (A); OipA (B). The concentration of IFN-γ (pg/mL) was expressed as a mean of three independent experiments ± SD. Statistically significant differences with the negative control are indicated as: ****(P<0.0001); ***(P=0.0001).

 ELISA test was used to determine the amounts of key cytokines (IFN-γ and IL-4). We observed that after 48 hours of incubation with OipA and propolis, the amounts of IL-4 and IFN-γ were significantly increased, which seemed to be concentration-dependent (P<0.0001). The optimum concentration of OipA, resulting in an increased secretion of IL-4, was 5μg/mL. At higher concentrations, the amount of IL-4 was progressively diminished until its production was completely suppressed at 40 μg/mL (Figure-4A). The optimum concentration of propolis, which results in the most significant increased secretion of IL-4, was 40 μg/ml (Figure-4B). The optimum concentration of OipA for induction of IFN-γ was 10 μg/ml but the production of IFN-γ was diminished with the higher concentrations of OipA until its basic production (Figure-5A). The optimum concentration of propolis, which increases the secretion of IFN-γ, was also 40 μg/mL (Figure-5B). Also, the amount of IL-4 was significantly increased at a lower concentration of 10 μg/mL (Figure-4B).

## Discussion

Developing a prophylactic and/or therapeutic vaccine requires an immunogenic bacterial antigen and a combination of suitable antigen with an effective adjuvant and the most eligible administration route [24]. Numerous investigators have evaluated antigenic properties of *H*. *pylori *virulence factors such as urease, catalase, VacA, CagA, NapA, GroES, AlpA, BabA, HpaA, SOD, and OipA recombinant proteins to obtain a protective vaccine against *H*. *pylori *infection [25,26]. The regulatory roles of Type 1 and 2 helper T cells (Th1 and Th2) in immune protection against *H. pylori *infection are partly understood. The evaluation of the proper impact for such a study would be the absence of a comparable animal model as humans. The absence of an *in *vitro/in situ evaluation model may also increase the impact of such evaluation. Concerning its evaluation in mice, Akhiani *et al*. [25] demonstrated that Th1 cells and the cytokines IFN-γ and IL-12 are crucial for developing immune protection against *H. pylori *infection. Due to the unique immunity mechanism against *H. pylori* infection, it is important to understand the kind of immunity the recombinant protein, as immunizer, would be triggered. We not only have evaluated OipA as an antigen but also the effects of its regulatory amounts in the induction of IL-4 and IFN-γ. We found that OipA recombinant protein could affect both IL-4 and IFN-γ production in macrophages, but the stimulating concentration that releases the cytokines was different. According to previous studies, Th1 cells are responsible for protective immunity against *H. pylori* [25]. Indeed, we need to activate the Th1 immune system pathway for protection against *H. pylori*. IFN-γ is the critical cytokine in Th1 response immunity, and its increase by an immunogenic protein explained that Th1 cytokines cascade responses might be activated. It was also found that an increment in IFN-γ and IL-4 secretion elicited by OipA was distinct, which will be indicated by a mixed Th1/Th2 response. Moreover, it is observed that an OipA concentration of 10 μg/mL may also be effective in immunization because it was more effective due to its stimulation of IFN-γ production, a concentration that did not stimulate high IL-4 production. In the study reported by Chen *et al.* [27], the same antigen was evaluated to be the most effective. For this purpose, an OipA encoding DNA construct was used to vaccinate C57BL/ 6 mice, which resulted in reduced colonization by *H. pylori* [27]. They found that IL-2 and the B subunit encoding DNA of heat-labile toxin of *E. coli*, as an adjuvant, had a positive modulation of immune response to the Th1 effectors’ immune response in mice. In another investigation, Chen *et al*. used *Salmonella typhimurium* to express an optimized *oipA* for vaccination [28]. They also found that oral administration of the OipA DNA vaccine to mice caused significantly higher levels of IgG2a/IgG1 antibodies and IFN-γ/IL-4 cytokines, indicating a mixed Th1/Th2 immune response and decreased bacterial colonization in vaccinating mice [29]. In the last evaluation, researchers have also found a similar effect of OipA as a vaccinating antigen [23]. They used propolis as adjuvant and OipA recombinant protein as antigen in the C57BL/6 mice model and showed the IgA titers were significantly higher in the OipA group compared to the control. However, they could not find an adjuvant effect of propolis in their study [23]. In the present study, we demonstrated that OipA recombinant protein could modulate Th1-related immune response, while IFN-γ may be significantly increased in a specific concentration of OipA in comparison with Il-4. Achieving a proper immune response by an immunogenic antigen would be the key to a successful immunization. The use of propolis in the present study was based on its potential adjuvant activity because it was proposed that propolis improves humoral and cellular immune responses, especially Th1-related immune response [13,30-31]. In this study, the roles of various propolis concentrations were tested in the induction of two key cytokines. We observed at higher concentrations of propolis (40 μg/ml), the IL-4, as well as IFN-γ production, was elicited, whereas the production of both IFN-γ and IL-4 was repressed at lower concentrations. Therefore, observation of unexpected adverse effects of propolis towards OipA in mouse during the last works [23] may be perhaps due to their inappropriate concentrations. In higher concentrations, the phenolic, flavonoid, or other compounds may attach to OipA and partially affect its antigenic structure. This likelihood may be prevented by selecting a better concentration of OipA and propolis, which may favor the production of IFN-γ in animals [15,32,33]. Consequently, the hypothesis of the selective immunogenic effect of propolis in triggering the macrophage cells was proven to produce IFN-γ. However, the effective concentration of propolis for inducing the maximum secretion of IFN-γ was 40 μg/ml, which was the same as IL-4 production. This may be the cause of the mixed response of Th1/Th2, which can influence the effective response of OipA in immunization. The high concentration of propolis along with a low concentration of OipA could be a reason for the adverse effects of propolis in the previous study [23].

## Conclusion

 In this study, a separate evaluation was conducted for the immunogenic effect of propolis and OipA on the macrophage cells. Firstly, the potential ability of the OipA as an antigen and the propolis as an adjuvant was investigated, and secondly, their effective amount or dose for the immune system stimulation was evaluated. Despite in vivo complexities, further study in an animal model is separately and simultaneously proposed to evaluate the effective concentrations of OipA and propolis, the adjuvant property of the propolis and its effect on the immune system.

## Conflict of Interest

 The authors have no substantial financial or commercial conflicts of interest with the current work or its publication.
